# Surgical setting of initial cholecystectomy influences prognosis in incidentally detected gallbladder carcinoma

**DOI:** 10.3332/ecancer.2026.2073

**Published:** 2026-02-05

**Authors:** Ajay Kumar Yadav, Anshuman Pandey, Rahul Singh, Hareesh Shanthappa Nellikoppad, Bhanu Pratap Singh

**Affiliations:** Department of Surgical Gastroenterology, Dr. Ram Manohar Lohia Institute of Medical Sciences, Lucknow 226010, India

**Keywords:** incidental gall bladder cancer, gall bladder cancer, tertiary centre, peripheral centre, laparoscopic cholecystectomy

## Abstract

**Background:**

Gallbladder cancer (GBC) is a common biliary malignancy in India, with many cases diagnosed incidentally as gallbladder carcinoma (IGBC) after cholecystectomy for presumed benign disease. IGBC often has a better prognosis due to earlier stage detection. However, limited access to high-quality imaging, especially in peripheral centres, contributes to missed preoperative diagnoses. Routine histopathological examination has improved detection rates. This study aims to compare survival outcomes and identify prognostic factors in IGBC patients treated at peripheral versus tertiary care centres.

**Methods:**

This retrospective study reviewed medical records of patients diagnosed with IGBC following cholecystectomy for presumed benign disease between 2015 and 2022 at a North Indian tertiary hospital. Patients were grouped based on initial surgery site (tertiary versus peripheral centres). Management followed National Comprehensive Cancer Network guidelines, with T1a patients advised follow-up and T1b or higher undergoing radical resection. Data on clinical, pathological and treatment parameters were analysed. Survival outcomes were assessed using Kaplan–Meier and Cox regression methods.

**Results:**

Of 39 IGBC patients reviewed, 30 were included in the final analysis. No significant differences were observed between tertiary and peripheral groups in demographics, symptoms or pathology. However, patients from peripheral centres had longer delays between surgeries and higher rates of metastasis. Overall survival was significantly better in the tertiary group (3- and 5-year OS: 88.2% and 72.8%) compared to the peripheral group (53.6% and 26.9%, *p* = 0.01). Multivariate analysis did not identify significant independent prognostic factors.

**Conclusion:**

Patients undergoing cholecystectomy at peripheral centres for presumed benign disease and later diagnosed with IGBC have poorer survival, primarily due to delayed diagnosis, limited resources and late referral. In contrast, timely referral, standardised evaluation and specialised care at tertiary centres significantly improve survival outcomes.

## Introduction

Gallbladder cancer (GBC) is one of the most common malignancies of the biliary tract, with a notably high incidence in India [1, 2]. GBC identified incidentally on histopathological examination following cholecystectomy for presumed benign disease is termed incidental gallbladder carcinoma (IGBC). The incidence of IGBC ranges from 0.14% to 1.6%, and it is higher in endemic regions such as India. IGBC generally has a better prognosis than GBC diagnosed preoperatively, likely due to its detection at earlier disease stages [3–6].

Clinical presentations of GBC and cholelithiasis are similar and there are no reliable biochemical markers for diagnosis of GBC [7–14]. Proper preoperative imaging by an expert radiologist is key in differentiating benign and malignant gallbladder disease and thus reducing the incidence of IGBC. However, in early disease, imaging—especially ultrasound examination—often fails to detect any abnormality such as asymmetric wall thickening, particularly when associated with cholelithiasis [15]. Contrast enhanced computed tomography can differentiate neoplastic and benign gallbladder wall thickening but is not routinely done, particularly in developing countries [16, 17].

Managing patients with IGBC includes complete staging with chest and abdominal computed tomography (CT) and/or positron emission tomography (PET)-CT followed by surgery [18–23]. Surgical management of IGBC depends on the tumour stage, with T1a lesions requiring no additional surgery beyond cholecystectomy, while T1b and more advanced stages often necessitate reoperation with a radical cholecystectomy [24–26]. Optimal timing for reoperation is ideally within 4 weeks after the initial surgery, following comprehensive restaging, as this window is associated with better outcomes [27].

The prognosis of IGBC depends on several factors, including tumour histology, T stage, lymph node involvement, lymphovascular and perineural invasion (PNI), surgical margin status and gallbladder perforation during the initial surgery [28–31].

In developing countries like India, where gallbladder disease is highly prevalent, many cholecystectomies for presumed benign conditions are performed at peripheral or less-experienced centres. These centres often have limited access to high-quality preoperative imaging and therefore often misdiagnose GBCs as benign disease. However, routine histopathological examination of all gallbladder specimens, even in these peripheral settings, is becoming increasingly common. These factors have contributed to a rising detection rate of IGBC.

This study aims to evaluate the differences in survival outcomes of patients with IGBC who underwent cholecystectomy at peripheral centres compared to those treated at tertiary care institutions. Additionally, it seeks to identify potential prognostic factors that may influence survival in patients diagnosed with IGBC.

## Materials and methods

This retrospective study was conducted at a tertiary care teaching hospital in North India. Medical records of patients who underwent cholecystectomy for presumed benign gallbladder disease between 2015 and 2022, either at the tertiary care institute or at peripheral hospitals and were later diagnosed with IGBC were reviewed. Peripheral centres were defined as hospitals which does not have have multi-specialty facilities and rely on other hospitals for imaging and histopathological examinations while the tertiary centres was defined as institution with multispeciality facilities including imaging and histopathological examinations in the same centre. IGBC was defined as GBC that was not suspected based on preoperative clinical or radiological evaluation and was identified only on postoperative histopathological examination. All procedures were carried out in accordance with institutional and national ethical standards, and the study was approved by the Institutional Ethics Committee (IEC No. 104/23).

Evaluation and further management of patients after diagnosis of IGBC were as per National Comprehensive Cancer Network guidelines. Histopathological slides and reports from peripheral centres were reviewed by institutional pathologists for confirmation of diagnosis. Patients diagnosed with T1a disease were advised routine follow-up, whereas those with T1b or higher-stage disease underwent staging laparoscopy followed by radical cholecystectomy.

Data collected included demographic characteristics, presenting symptoms, preoperative diagnosis, biochemical parameters prior to the primary cholecystectomy, intraoperative findings during initial cholecystectomy, and the time interval between the initial surgery and presentation at the tertiary centre. Additional variables recorded were the duration between the initial and definitive surgery (i.e., radical cholecystectomy), and final histopathological details were recorded after definitive surgery such as tumour T and N stage, histological differentiation, surgical margin status, tumour type, presence of lymphovascular invasion (LVI), PNI and the longitudinal anatomical location of the tumour within the gallbladder. Patients were categorised into two groups based on the site of initial surgery: the tertiary centre group and the peripheral centre group. All patients were followed up either through outpatient visits or telephonic communication until 31 December 2024 or until death.

Statistical analysis was done using GraphPad Prism V 10.5.0. Categorical variables were compared using the chi-square or Fisher’s exact tests. Continuous variables were summarised as mean and standard deviation and were compared using the student *t*-test or analysis of variance. Primary endpoints of the study was overall survival (OS) and was analysed using Kaplan–Meier estimates. Survival curves were calculated using the log-rank test. Multivariate analysis using cox regression was done to identify the independent prognostic factors. A *p* value of < 0.05 was considered to be statistically significant.

## Results

A total of 39 patients who presented to the outpatient department with IGBC were reviewed. Of these, nine patients were excluded from the study: seven due to incomplete medical records and two due to loss to follow-up. Thus, 30 patients with IGBC were included in the final analysis.

**Figure 1.**
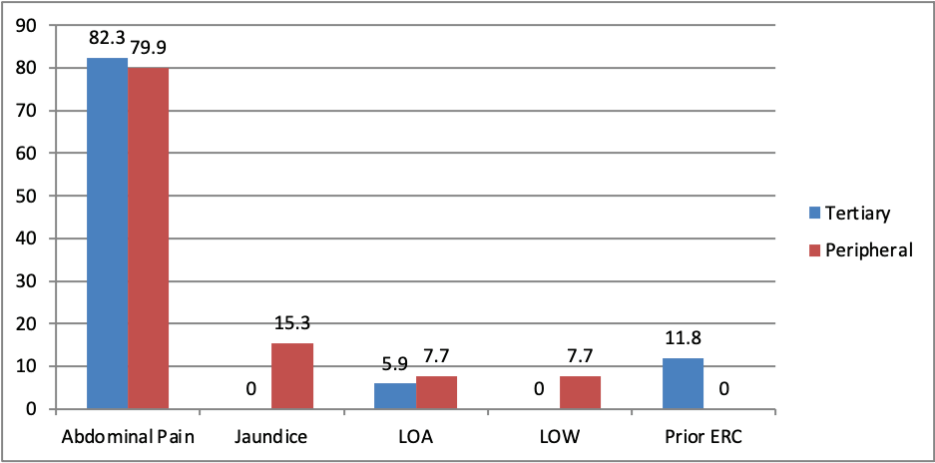
Clinical presentation of patients in two groups. LOA: Loss of appetite, LOW: Loss of weight.

There were no statistically significant differences between the two groups with respect to gender distribution, age, history of abdominal pain, jaundice, weight loss or loss of appetite (Figure 1). Abdominal pain was the most common presenting symptom in both groups, reported in approximately 80% of patients.

No significant differences were observed between the two groups based on biochemical evaluations or imaging findings. Notably, all patients in the tertiary centre group underwent routine evaluation of tumour markers (CEA and CA 19-9), whereas none of the patients in the peripheral centre group received this evaluation. Initial evaluation for preemptive diagnosis of gall stone disease was done with ultrasound scan of abdomen. Additional imaging was performed in eight patients of tertiary center group and three patients in peripheral center group underwent CECT abdomen for reasons not known. The details of imaging done in the peripheral centre group was not available. However, following clinical and radiological evaluation, all patients were diagnosed with benign biliary disease and no preoperative suspicion of malignancy was identified based on the available imaging and workup at respective centres (Table 1).

### Intra-operative findings

**Figure 2.**
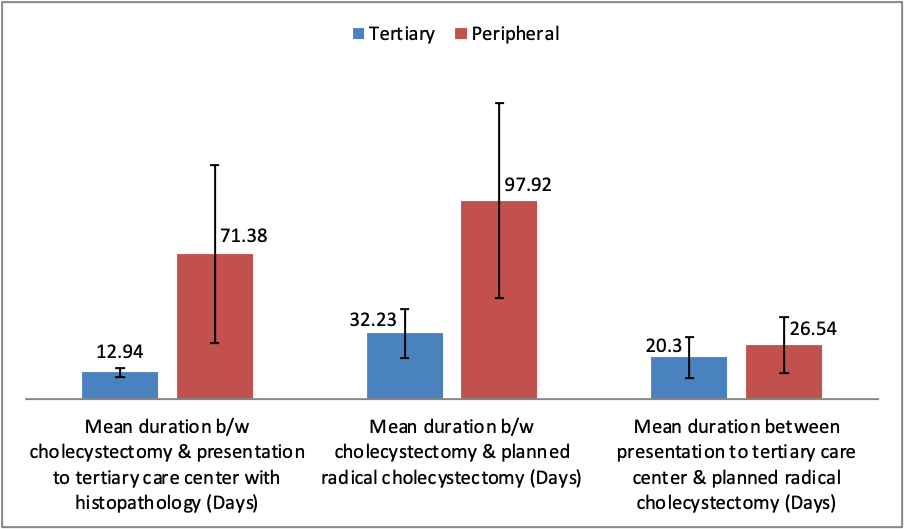
Comparison of mean duration at presentation to tertiary care institute and radical surgery between two groups.

All patients in tertiary center group were planned for laparoscopic cholecystectomy, 11.8% (*n* = 2) required conversion to open in view of dense adhesions and one patient underwent subtotal cholecystectomy. In peripheral center group, 46.1 % (*n* = 6) underwent laparoscopic cholecystectomy, 15.4 % (*n* = 2) required conversion to open, while 38.5% (*n* = 5) underwent upfront open cholecystectomy. Intraoperative details were available only in seven patients of peripheral center group. There was a significant difference (*p* = 0.003) in the intra-operative finding of gall bladder perforation and bile spillage between the two groups. While none in tertiary center group, 42.8% (*n* = 3 of 7) patients in peripheral center group had intra-operative gall bladder perforation. 17.6% patients (*n* = 3) in tertiary center group were suspicious of malignancy. Intraoperative frozen was not available in two patients and they did not consent for anticipated radical cholecystectomy (Figure 2). One patient was negative for malignancy on frozen section. 14.3% (*n* = 1 of 7) patient who underwent cholecystectomy at peripheral center intraoperatively was found suspicious of malignancy on cut section and was referred to our institute for further management (Table 1).

After presentation with histopathology findings of IGBC, patients treated at the tertiary care centre significantly shorter durations between cholecystectomy and planned radical cholecystectomy compared to those treated at the peripheral centre. However, after presentation with histopathology there was no significant delay between two groups between presentation at tertiary centre and planned staging laparoscopy followed by radical cholecystectomy. There was significantly higher metastasis observed in the peripheral centre group compared to tertiary centre group (Table 2). No patient had in hospital mortality following radical resection.

There was no significant difference in histopathological type, T-stage, tumour differentiation and longitudinal anatomical location, resection margin, LVI and PNI in both groups (Table 3). Most common stage of presentation in both groups was T2 (52.9%); however, more patients (23.1%) in peripheral centre group presented with T3 stage as compared to tertiary centre group (11.8%).

### Survival outcome

**Figure 3.**
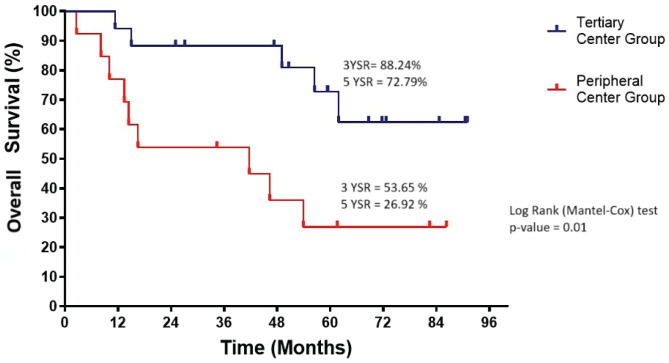
Overall survival of tertiary and peripheral centre group (*n* = 30). YSR, year survival rate.

The OS in the tertiary centre group was significantly better than in the peripheral centre group (*p* = 0.01) (Figure 3). The 3-year and 5-year survival rates were 88.24% and 72.79%, respectively, in the tertiary centre group, compared to 53.65% and 26.92% in the peripheral centre group.

### Factors associated with survival in patients with gall bladder cancers

Since we confirmed that peripheral centre group has poorer survival outcome, multivariate analysis was carried out using a least squares regression to determine the factors influencing the OS. The regression model does not demonstrate statistical significance, as indicated by the high *p*-values for the *F*-statistic and individual predictors. While the model explains a substantial portion of the variance (*R*² = 0.7402), the lack of significant predictors suggests that other unmeasured factors may be influencing the dependent variable. Additionally, the moderate multicollinearity and small sample size may impact the robustness of the findings.

## Discussion

Worldwide, the incidence of IGBC ranges between 0.2% and 3%, and in endemic countries like India, the reported incidence for IGBCs is higher [32, 33]. The probable reason explaining this higher incidence is that most evaluations and cholecystectomies performed for benign gallbladder diseases are done at centres without multidisciplinary units and with limited resources, thus increasing the incidence of GBC diagnosed on histopathology (IGBC). IGBC has a better prognosis than GBC diagnosed preoperatively, likely due to its detection at earlier disease stages [5, 6]. Wherever there is any suspicion of neoplastic disease during ultrasonography (USG), a careful discussion should be made with an expert radiologist and at least a cross-sectional imaging (CT/magnetic resonance imaging should be done as per current guidelines. The radiological evidence of GBC can be challenging to interpret, as it can present with non-specific findings such as gallbladder wall thickening, which can lead to confusion with other conditions like chronic cholecystitis [17]. Biochemical markers (CEA, CA 19-9) do not have a definitive role in the diagnosis of GBC; however, in cases with thick-walled gallbladder, they may aid not only in diagnosis but also in postoperative follow-up [13, 14, 34]. Newer studies have shown optimal cut-off values to predict the prognosis of GBC to be >5 IU/mL for CEA and >65 IU/mL for CA 19-9, with moderate sensitivity and high specificity [35]. At our institute, we routinely assess serum CEA and CA 19-9 levels in gallbladder disease due to the high incidence of GBC in North India. In the present study, cross-sectional imaging was performed in eight patients (47.1%) of the tertiary centre group. Two patients with raised bilirubin levels underwent MRI, which revealed choledocholithiasis, and were managed by initial endoscopic retrograde cholangiography and stone removal followed by interval cholecystectomy, while CECT abdomen was done in six patients with wall thickness >3 mm or raised CA 19-9 levels. In contrast, only three patients (23.1%) in the peripheral centre group underwent CECT abdomen for unclear indications. This is notable, as five patients had wall thickness >3 mm on USG, and two had raised serum bilirubin levels—suggesting inadequate preoperative evaluation in this group. Although these differences were not statistically significant, they may indicate a higher rate of missed GBCs with poorer survival outcomes in the peripheral centre group, rather than true IGBCs.

Inadvertent bile spillage during cholecystectomy has negative implications for the prognosis of GBC, as potential dispersal of cancer cells in the abdomen can lead to peritoneal carcinomatosis, contributing to a worse outcome [36, 37]. Therefore, it is vital for surgeons to approach these procedures with extreme caution to improve patient safety and outcomes. This highlights the importance of meticulous surgical techniques and careful management to prevent unintended consequences on prognosis. In the present study, intraoperative gallbladder perforation was significantly (*p* = 0.003) higher in the peripheral centre group, indicating that surgeries were performed with limited resources, which might explain the poorer survival in patients operated on at peripheral centres. However, on multivariate analysis, intraoperative gallbladder perforation was not found to be an independent risk factor for poor survival.

The management of incidental GBC, after thorough preoperative assessment including cross-sectional imaging and biochemical marker analysis, involves oncological resection in the form of radical cholecystectomy for stages T1b and above. In cases where there is intraoperative suspicion of GBC, an anticipated radical cholecystectomy should be performed. If expertise for this procedure is not available at the current facility, early referral to a higher centre with specialised capabilities should be arranged. This approach is essential to ensure optimal care and outcomes for individuals with GBC [25]. In this study, three patients who underwent cholecystectomy at our institute were suspicious for malignancy on cut section. However, intraoperative frozen section was not available in two patients and they did not consent for anticipated radical cholecystectomy. One patient was reported negative for malignancy on frozen section. Only a single patient who underwent cholecystectomy at a peripheral centre was intraoperatively found suspicious for malignancy on cut section and was referred to our institute.

The optimal timing for completion surgery following initial cholecystectomy in IGBC remains undefined; however, current literature suggests that reoperation should ideally be performed within 4–8 weeks post-cholecystectomy, after resolution of postoperative inflammation. Delays beyond 8 weeks are associated with poorer survival outcomes, likely due to disease progression and dissemination [27]. Early surgical intervention requires timely referral to a higher centre. This is reflected in the present study, where patients who underwent initial cholecystectomy at peripheral centres experienced a significant delay in referral to tertiary care following histopathological diagnosis of IGBC (*p* < 0.0001). This delay may be attributed to the lack of structured postoperative follow-up and inadequate communication with patients. As a result, a significantly higher proportion of patients in the peripheral centre group (53.8%) were found to have metastatic disease during staging laparoscopy or re-exploration, compared to the tertiary centre group (*p* = 0.0007). This likely contributed to the poorer OS observed in the peripheral centre group. There was no significant delay between the two groups in completion surgery after presentation to our institute (Table 2).

The histopathological findings in this study were consistent with existing literature in terms of histological type, T-stage distribution, tumour differentiation, anatomical location within the gallbladder, as well as LVI and PNI status [4, 38]. Although not statistically significant, more patients (23.1%) in peripheral centre group had evident tumour on gross examination as compared to tertiary centre (5.9%) group indicating missed GBC rather than true IGBCs. Adenocarcinoma was the most common histological type observed in both groups. The predominant T-stage was T2 in both groups, and the most frequent tumour location was the fundus, followed by the body of the gallbladder. Although not statistically significant, a higher proportion of patients in the peripheral centre group had T3 disease (23%) compared to the tertiary centre group (11%). The rate of nodal positivity was similar between groups, with 17.6% in the tertiary centre and 23.1% in the peripheral centre (*p* = 0.71). The mean number of lymph nodes sampled was higher in the peripheral group (5.5 ± 1.87) compared to the tertiary group (4.06 ± 2.61; *p* = 0.10). The lymph node ratio (LNR) was lower in the tertiary group (0.04 ± 0.08) than in the peripheral group (0.12 ± 0.15; *p* = 0.07), suggesting better oncological outcomes. Optimal histopatholgical details like tumour location, tumour focality, background associated lesions like chronic cholecystitis, margin status were missing in peripheral centre reporting, reflecting suboptimal evaluation and possibly contributing to under-staging and poorer survival outcomes.

These finding suggests a greater likelihood of missed GBCs, rather than true incidental GBC (IGBC), in the peripheral centre group. Such cases might have been detected preoperatively through appropriate cross-sectional imaging, tumour marker evaluation and the use of intraoperative frozen section analysis. Given that missed GBC, as opposed to true IGBC, is associated with poorer survival outcomes [39], these likely contributed to the inferior OS observed in patients from the peripheral centre group.

In GBC, adjuvant therapy can be considered in patients with incomplete resection (R1 or R2) and more advanced disease, particularly in cases with nodal involvement [40]. In this study, two patients with T3 disease in the tertiary centre received adjuvant chemotherapy. In the peripheral centre group, one patient received adjuvant chemotherapy after curative resection and three patients with metastatic disease received palliative chemotherapy.

This study has several limitations, including its retrospective design and small sample size, which limit the generalisability of the findings. Additionally, unmeasured confounding factors may have influenced the results, as multivariate analysis did not identify statistically significant prognostic predictors. Variability and incomplete data from peripheral centres may have affected the accuracy of preoperative and intraoperative information. Furthermore, limited access to intraoperative frozen section in some cases could have impacted surgical decision-making and outcomes. Despite these limitations, this study is one of the first from an endemic region to compare IGBC outcomes between peripheral and tertiary healthcare settings, offering important insights into how differences in resource availability and referral patterns affect patient survival and management in such contexts.

## Conclusion

This study highlights the significant impact of the initial treatment setting on survival outcomes in patients diagnosed with IGBC. Patients who underwent cholecystectomy at tertiary care centres demonstrated significantly better OS compared to those treated at peripheral centres. This difference is likely attributable to more timely referral, comprehensive preoperative evaluation and better intraoperative surgical practices at tertiary centres. Delays in diagnosis, inadequate preoperative assessment and higher rates of intraoperative gallbladder perforation in peripheral centres contributed to a higher incidence of advanced-stage disease and metastasis at the time of completion surgery. These findings underscore the need for heightened clinical suspicion, adherence to standard diagnostic protocols and timely referral to specialised centres to improve outcomes in IGBC. To overcome these limitations, future prospective studies with larger sample sizes are warranted to strengthen the evidence base and minimise selection bias. Standardised data collection and improved coordination between peripheral and tertiary centres would enhance data quality and consistency.

## List of abbreviations

AJCC: American Joint Committee on Cancer, CA 19-9: Carbohydrate Antigen 19.9, CBD: Common Bile Duct, CEA: Carcino-Embryonic Antigen, CECT: Contrast enhanced Computed Tomography, ERCP: Endoscopic Retrograde Cholangiopancreatography, GBC: Gallbladder carcinoma, IGBC: Incidental Gall Bladder Carcinoma, IHBRD: Intrahepatic Biliary Radicle Dilatation, LVI: Lymphovascular invasion, MRCP: Magnetic Resonance Cholangiopancreatography, MRI: Magnetic Resonance Imaging, NCCN: National Comprehensive Cancer Network, OS: Overall Survival, PET: Positron Emission tomography, PNI: Perineural invasion.

## Conflicts of interest

The authors have no conflicts of interest to disclose.

## Funding

This research did not receive any specific grant from funding agencies in the public, commercial or not-for-profit sectors.

## Author contributions

Study conception and design: Ajay Kumar Yadav designed the study and wrote the manuscript. Acquisition of data: Ajay Kumar Yadav. Data analysis and interpretation: Ajay Kumar Yadav. Critical revision of the manuscript: Anshuman Pandey. All authors approved the final manuscript.

## Figures and Tables

**Table 1.** Comparison of demographic details, history, biochemical parameters, radiological parameters between two groups.

	Tertiary centre (*n* = 17)	Peripheral centre (*n* = 13)	*p* value
Male/Female (***n***)	5/12	2/11	0.37
Age (Years)	54.82 ± 14.64	49.69 ± 9.88	0.29
**History**			
Pain abdomen **(%**)	82.3 (*n* = 14)	79.9 (*n* = 10)	0.71
Jaundice **(%**)	0	15.3 (*n* = 2)	0.09
Loss of weight (***n***)	0	7.7 (1)	0.24
Loss of appetite (***n***)	5.9 (1)	7.7 (1)	0.84
Prior ERC (***n***)	11.8 (2)	0	0.2
**Biochemical parameters**			
Bilirubin (>2 mg/dL)** (%**)	11.7 (*n* = 2)	15.4 (*n* = 2)	0.77
CEA (>5 ng/mL) (***n***)	0	-	
CA 19.9 (>30 U/mL)** (%**)	5.9 (*n* = 1)	-	
**Radiological parameters**			
**Ultrasound**			
Size of stone (>2.5 cm) (%)	2/14 (14.3%)	2 (15.4%)	
Wall thickness (>3 mm) (%)	35.3 (*n* = 6)	38.5 (*n* = 5)	0.86
**Pre-op CT/MRI** (%)	47 (*n* = 8)	23 (*n* = 3)	0.18
**Pre-operative diagnosis**			
Gall stone disease	64.7 (*n* = 11)	84.6 (*n* = 11)	0.7
**Thick walled gall bladder**	11.8 (*n* = 2)	7.7 (*n* = 1)	
**Polyp**	17.6 (*n* = 3)	7.7 (*n* = 1)	
**GSD + Polyp**	5.9 (*n* = 1)	0	
**Procedure**			
Lap cholecystectomy (%)	88.2 (*n* = 15)	46.1 (*n* = 6)	
Lap to open cholecystectomy (%)	11.8 (*n* = 2)	15.4 (*n* = 2)	
Open cholecystectomy (%)	0	38.5 (*n* = 5)	
Subtotal cholecystectomy	5.9 (*n* = 1)	15.4 (*n* = 2)	0.4
Total cholecystectomy	94.1 (*n* = 16)	84.6 (*n* = 11)	
**Intra-operative findings**			
Gall bladder perforation (%)	0	3/7 (42.8%)	0.003
Cut section suspicious (%)	17.6 (*n* = 3*)	1/7^@^ (14.3%)	0.84

* Three patients who underwent cholecystectomy at our institute were suspicious of malignancy. Intraoperative frozen was not available in two patients and did not consent for anticipated radical cholecystectomy. One patient was negative for malignancy on frozen section

@ One patient who underwent cholecystectomy at peripheral centre intraoperatively was found suspicious of malignancy on cut section and was referred to our institute

**Table 2.** Comparison of mean duration of presentation to tertiary centre, timing of planned radical cholecystectomy and metastasis/unresectibility between two groups.

	Tertiary centre (*n* = 17)	Peripheral centre (*n* = 13)	*p*- value
Mean duration b/w cholecystectomy & presentation to tertiary care centre with histopathology (Days)	12.94 ± 2.38	71.38 ± 43.94	<0.0001
Mean duration b/w cholecystectomy & planned radical cholecystectomy (Days)	32.23 ± 12.29	97.92 ± 48.41	0.0001
Mean duration between presentation to tertiary care centre & planned radical cholecystectomy (Days)	20.30 ± 10.03	26.54 ± 13.74	0.2
Metastasis (%)*	0	53.8 (*n* = 7)	0.0007
Unresectable*	0	0	

* Metastasis/unresectibility identified intraoperatively at the time of planned radical cholecystectomy.

**Table 3.** Histopathological details of two groups.

	Tertiary centre (*n* = 17)	Peripheral centre (*n* = 13)	*p*- value
**Gross**			
Detectable	5.9 (*n* = 1)	23.1 (*n* = 3)	0.18
**Histological type**			
Adenocarcinoma (%)	100 (*n* = 17)	100 (*n* = 13)	
Others	0	0	
**T-stage (%)**			
T1a	23.5 (*n* = 4)	0	0.24
T1b	11.8 (*n* = 2)	23.1 (*n* = 3)	
T2	52.9 (*n* = 9)	53.8 (*n* = 7)	
T3	11.8 (*n* = 2)	23.1 (*n* = 3)	
T4	0	0	
**Differentiation (%)**			
Well	58.8 (*n* = 10)	46.1 (*n* = 6)	0.11
Moderate	41.2 (*n* = 7)	30.8 (*n* = 4)	
Poor	0	23.1 (*n* = 3)	
**Location (%)**			
Fundus	47 (*n* = 8)	61.5 (*n* = 8)	0.5
Body	23.5 (*n* = 4)	7.7 (*n* = 1)	
Neck	23.5 (*n* = 4)	11.7 (*n* = 2)	
Multifocal	5.9 (*n* = 1)	11.7 (*n* = 2)	
**Margin status after initial cholecystectomy (%)**			
Positive	0	0	
Negative	100 (*n* = 17)	46 (*n* = 6)	
Unknown	0	54 (*n* = 7)	
**Lymphovascular invasion (%)**			
Present	5.9 (*n* = 1)	23.1 (*n* = 3)	0.18
Absent	94.1 (*n* = 16)	76.9 (*n* = 10)	
Unknown	0	0	
**Perineural invasion (%)**			
Present	11.8 (*n* = 2)	15.4 (*n* = 2)	0.77
Absent	88.2 (*n* = 15)	84.6 (*n* = 11)	
Unknown	0	0	
**Node positive after definitive surgery**	17.6 (*n* = 3)	23.1 (*n* = 3)	0.71
**Nodes sampled (Mean ± SD)**	4.06 ± 2.61	5.5 ± 1.87	0.10
**LNR (Mean ± SD)**	0.04 ± 0.08	0.12 ± 0.15	0.07

LNR: lymph node ratio, SD: standard deviation
